# Atresia of the Ascending Colon: A Rarity

**DOI:** 10.1186/1752-1947-4-3

**Published:** 2010-08-14

**Authors:** Haroon Mansoor, Naila Kanwal, Mahmood Shaukat

**Affiliations:** Department of Pediatric Surgery, Mayo Hospital Lahore, Pakistan

**Keywords:** Atresia of ascending colon, Neonatal intestinal obstruction, Early ileostomy reversal

## Abstract

Atresia of the colon is among the rare types of all gastrointestinal atresias. Ascending colon is the rarest site of all the colonic atresias. The authors report a case of a 4-day-old male baby who presented with the features of distal intestinal obstruction. At laparotomy type I atresia of the ascending colon, just distal to cecum, was found. Primary ceco-colic anastomosis along with a covering ileostomy was performed. Ileostomy was reversed 3 weeks later.

## INTRODUCTION

Atresia of the ascending colon is one of the rarest causes of neonatal intestinal obstruction. Reported incidence of colonic atresia is 1 in 20,000 live births of which ascending colon is the rarest [1].

Due to rarity of the disease large series have not been reported in the literature. Colonic atresia occurs in descending order of frequency at sigmoid, splenic flexure, hepatic flexure and ascending colon respectively [2].

It may be associated with anomalies of other systems. Mortality is usually high when treatment is delayed for more than 72 hours. However prognosis is satisfactory with early diagnosis and proper management [3]. Rarity of the disease prompted the authors to report this case.

## CASE REPORT

A 4-day-old term male baby was born through cesarean section, to an otherwise healthy primigravida, at a peripheral private clinic. No prenatal problem was detected on routine antenatal visits.

The baby did not pass meconium till 4th day when he developed marked abdominal distension along with other features of intestinal obstruction. At the time of admission to our hospital along with distension mild dehydration was also present. There was no other apparent associated anomaly. Rectal stimulation was inconclusive.

Plain x-ray of the abdomen in erect posture showed multiple air fluid levels suggestive of distal small bowel obstruction. A diagnosis of distal small bowel atresia was made. Baby was optimized by fluid and electrolytes replacement. Parenteral antibiotics along with vitamin K were administered.

Laparotomy was performed through a right upper transverse incision. Operative finding was type I atresia of the ascending colon just distal to hugely distended cecum (Fig. 1, 2). A ceco-colic anastomosis was performed by opening the lumens of dilated cecum and micro ascending colon through a longitudinal incision on their anterior walls. Intervening mucosal septum was excised and the defect closed transversely with covering ileostomy. The recovery was smooth and patient discharged on 7th postoperative day. After 3 weeks a contrast radiograph through ileostomy was performed to confirm the distal patency before ileostomy reversal (Fig. 3).

**Figure:**
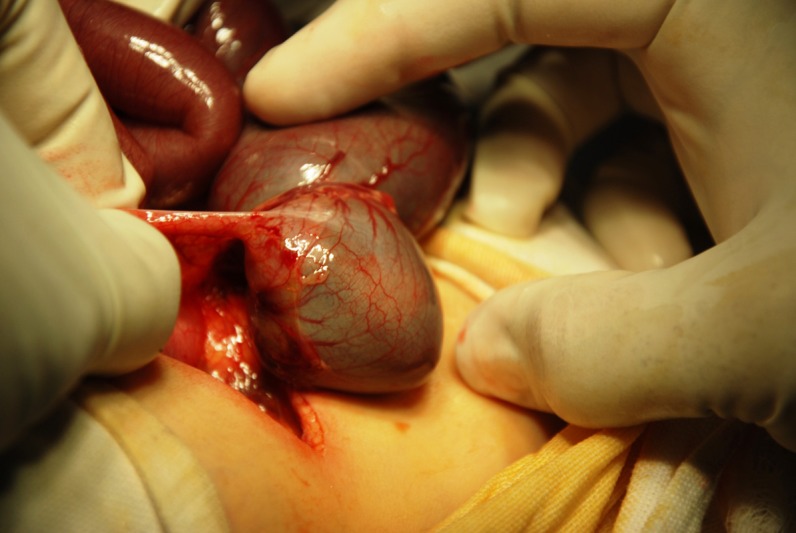
Figure 1: Atresia of the ascending colon with hugely distended cecum.

**Figure:**
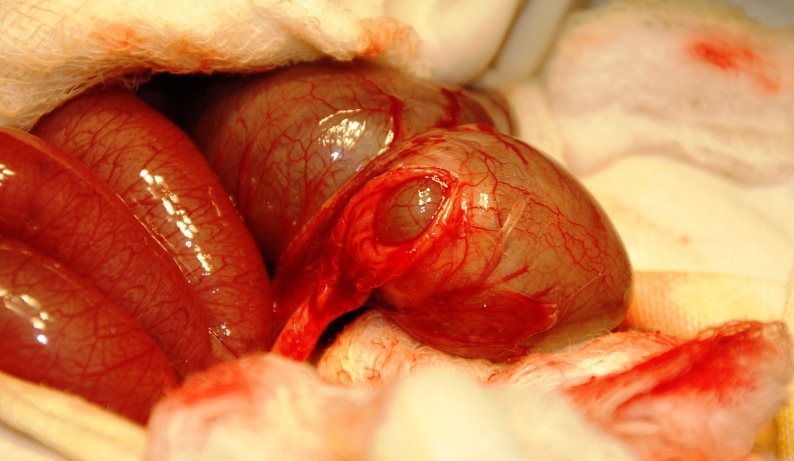
Figure 2: Mucosal septum of type I atresia and narrow lumen of ascending colon.

**Figure:**
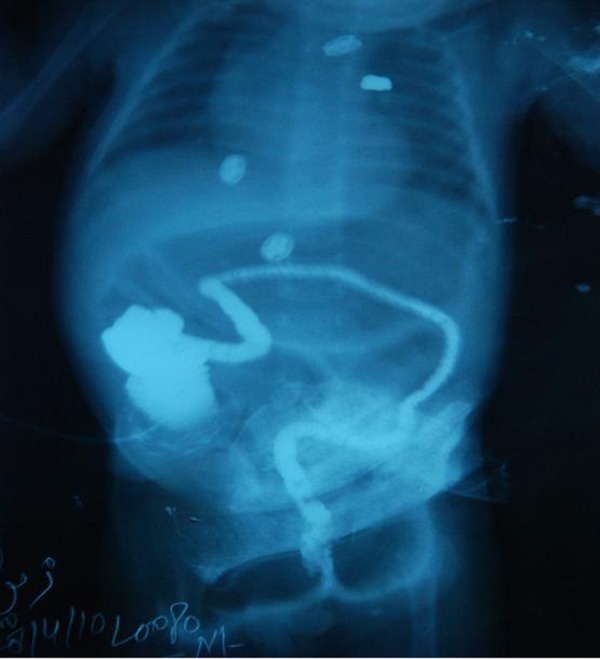
Figure 3: Contrast radiograph through ileostomy showing cecum and narrow lumen of unused distal colon.

## DISCUSSION

Colonic atresia accounts for 1.8-15% of intestinal atresias [4]. Ascending colon is the rarest site of colonic atresia. Due to its rarity it is usually not thought of in the differential diagnosis of neonatal intestinal obstruction [5].

Delayed recognition of symptoms increases the risk of complications like perforation and sepsis. Etiology of this anomaly is still debated. Commonly accepted theory is that of in-utero vascular accidents in the early gestation [6]. Colonic volvulus, intussusception, incarceration and strangulation of internal hernias in-utero, are also the probable etiological factors. Failure of recanalization after the solid cord stage as in duodenal atresia is also considered to be the cause of colonic atresia. Due to the rarity of the disease available literature is scanty. Associated anomalies like abdominal wall defects (gastroschisis) [7], musculoskeletal disorders, small gut atresia, ocular and facial anomalies are common [8].

Colonic atresia in babies with gastroschisis seems to result from the bowel compression with narrowing of abdominal defect [8].

Association of small bowel atresia and Hirschsprung’s disease is of paramount imporatance [9]. Rectal biopsy is recommended in patients who initially were treated for colonic atresia and had slow return of gut functions [10].

Uncomplicated right colonic atresia can be treated with primary anastomosis with little morbidity whereas staged reconstruction with proximal diversion is advised in sigmoid and left colonic atresia to avoid the complications of anastomosis [10].

Preservation of ileocecal valve is desired for future growth of the child. Due to hugely dilated cecum and the enormous disparity between the cecum and atretic ascending colon in the reported case primary cecocolic anastomosis with covering ileostomy seems appropriate. However the operative strategy depends on the clinical state of the patient and the safety of the procedure should always be a priority. In the case presented staged procedure was adopted and it resulted in early recovery and discharge of the patient. Stoma care is an issue in these cases especially with ileostomy where effluent is more fluid in nature. To address this issue an early reversal was performed in our patient. It thus appears to be an appropriate approach in a set up where stoma care may be an issue.

## Footnotes

**Source of Support:** Nil

**Conflict of Interest:** None declared
